# Efficacy and Safety of Teclistamab in Relapsed/Refractory Multiple Myeloma: 2‐Year Follow‐Up From MajesTEC‐1 China Cohort

**DOI:** 10.1002/jha2.70340

**Published:** 2026-07-30

**Authors:** Zhen Cai, Zhongjun Xia, Aili He, Yujun Dong, Yafei Wang, Aijun Liao, Yang Song, Hongye Chen, Athena Zuppa, Katherine Chastain, Latisha Watkins, Xinchao Luo, Lin Huang, Hongmei Xu, Longen Zhou, Wannan Chen, Angeline Zhu, Xiaohong Wang, Weijun Fu, Ting Niu, Juan Du

**Affiliations:** ^1^ First Affiliated Hospital Zhejiang University College of Medicine Hangzhou China; ^2^ Sun Yat‐sen University Cancer Center Guangzhou China; ^3^ The Second Affiliated Hospital of Xi'an Jiaotong University Xi'an China; ^4^ Peking University First Hospital Beijing China; ^5^ Tianjin Medical University Cancer Institute and Hospital Tianjin China; ^6^ Shengjing Hospital of China Medical University Shenyang China; ^7^ Johnson & Johnson Beijing China; ^8^ Johnson & Johnson, Raritan New Jersey USA; ^9^ Johnson & Johnson Spring House Pennsylvania USA; ^10^ Johnson & Johnson Singapore Singapore; ^11^ Shanghai Changzheng Hospital Naval Medical University Shanghai China; ^12^ West China Hospital Sichuan University Chengdu China

**Keywords:** bispecific antibody, MajesTEC‐1 Study, relapsed/refractory multiple myeloma, teclistamab

## Abstract

**Introduction:**

With an additional year of follow‐up compared with the previous report, this study presents the 27.2‐month efficacy and safety outcomes of the MajesTEC‐1 China cohort.

**Methods:**

A total of 26 participants with triple‑class exposed relapsed/refractory multiple myeloma (TCE‐RRMM) received subcutaneous teclistamab 1.5 mg/kg weekly, with potential transition to every 2 weeks in sustained responders.

**Results:**

Overall response rate (ORR) was 76.9%, with all responders achieving a very good partial response or better (≥ VGPR), and 75% achieving a complete response or better (≥ CR). Median progression‑free survival (PFS) was 25.1 months, while median duration of response (DOR) and overall survival (OS) were not reached. Among patients achieving ≥ CR, estimated 30‑month DOR, PFS, and OS rates were favorable at 80.0%, 80.0%, and 93.3%, respectively. Patients with poor prognostic features achieved ORRs comparable to overall population, and their DOR was generally consistent. Quality‐of‐life assessments showed improvements from baseline in global health status, physical functioning, fatigue, and pain. Safety profile was consistent with previous publication. Incidence of new‐onset Grade ≥ 3 infections decreased over time, which was consistent with the timing of transition to biweekly dosing and may be related to immunoglobulin use.

**Conclusion:**

These findings support teclistamab as a standard‐of‐care for patients with TCE‐RRMM in China.

**Clinical Trial Registration:**

ClinicalTrials.gov identifier: NCT03145181/NCT04557098

## 1 Introduction

Multiple myeloma (MM) is a cytogenetically heterogeneous clonal plasma cell malignancy that typically presents with the characteristic CRAB features, including hypercalcemia, renal impairment, anemia, and bone lesions [1]. The prevalence of MM in China has increased six‐fold from 1990 to 2021, with its incidence tripled and mortality rate doubled [2]. The current standard‐of‐care (SOC) therapies for MM include immunomodulatory drugs (IMiDs), CD38 monoclonal antibodies (anti‐CD38 mAbs), and proteasome inhibitors (PIs) [3, 4, 5]. As many patients eventually relapse or become refractory to these agents, leading to an emergent unmet medical need in triple‐class‐exposed relapsed or refractory MM (TCE‐RRMM) patients [6, 7, 8]. Approved therapies in China for TCE‐RRMM include B‐cell maturation antigen (BCMA)‐directed chimeric antigen receptor T‐cell (CAR‐T) therapies, such as ciltacabtagene autoleucel [9], equecabtagene autoleucel [10], zevorcabtagene autoleucel, and XPO‐1 inhibitor selinexor [11]. However, selinexor provides only modest clinical benefit, with reported response rate of ∼25% [12]. CAR‐T therapies, although highly active, are limited by accessibility challenges, including prolonged manufacturing times, apheresis requirements, and potential manufacturing failures. As a result, there remains an unmet need for more accessible and effective treatment options for patients with TCE‐RRMM in China [13, 14].

Teclistamab, a bispecific antibody (BsAb) targets BCMA and CD3, designed to redirect T‐cells to eliminate malignant plasma cells [15], has shown promising efficacy and manageable safety both in MajesTEC‐1 pivotal cohort and MajesTEC‐1 China cohort. In the MajesTEC‑1 pivotal cohort (*n* = 165; median follow‑up 30.4 months), teclistamab achieved an overall response rate (ORR) of 63.0%, with 46.1% of patients attaining complete response or better (≥ CR). Median duration of response (DOR), progression‐free survival (PFS), and overall survival (OS) were 24.0, 11.4, and 22.2 months, respectively, and all remained unreached among patients achieving ≥ CR. The clinical activity observed in the MajesTEC‐1 pivotal cohort has been further supported by data from the China cohort (*N* = 26), in which teclistamab demonstrated an ORR of 76.9% after a median follow‑up of 15 months. Rates of very good partial response or better (≥ VGPR) and ≥ CR were 76.9% and 57.7%, respectively. Among 20 patients with available minimal residual disease (MRD) samples, 18 (90%) were MRD negative 10^−5^ at any time point as detected by NGF. Among patients achieving ≥ CR with available MRD samples, 93.3% (14/15) achieved MRD negativity. At 12 months, the rate of DOR, PFS, and OS rates were 78.5%, 68.0%, and 83.5%, respectively [16]. With a 15‐month follow‐up, median DOR, PFS, and OS were not reached, and overall efficacy outcomes were consistent with those from the pivotal cohort [16]. While teclistamab showed a manageable safety profile, infections remain an important clinical consideration with teclistamab therapy. The incidence of infections was 78.8% in the pivotal cohort (Grade 3/4: 55.2%) and 96.2% in the China cohort (Grade 3/4: 69.2%) [16, 17]. These findings highlight the importance of a proactive infection‐mitigation strategy in patients receiving BCMA‐targeted BsAbs, particularly given the inherently increased infection risk associated with relapsed/refractory disease. This should include close clinical and laboratory monitoring, routine antimicrobial prophylaxis when appropriate, and immunoglobulin (IgG) replacement to maintain IgG levels above 400 mg/dL. With additional 12 months follow‐up compared to last report, this paper presents a 27.2‐month efficacy and safety analysis of MajesTEC‐1 China cohort, including more matured mPFS, subgroup analysis, additional analysis of patient‐reported outcomes (PROs), and a deeper dive in infection management and IgG use.

## 2 Methods

### 2.1 Study Design

MajesTEC‐1 (NCT03145181/NCT04557098) is a Phase 1/2, single‐arm, open‐label, multicenter study among adult participants with RRMM who had received ≥ 3 prior line of treatments (LOTs), including PI, IMiD, and anti‐CD38 mAbs [18, 19]. Following the pivotal cohort [19], a separate China cohort at the recommended Phase 2 Dose (RP2D) was conducted to evaluate the efficacy and safety of teclistamab. Consistent with the design of the pivotal cohort, the primary endpoint was ORR. The China cohort enrolled 26 participants from 7 centers between December 2021 and September 2022. Participants received 1.5 mg/kg teclistamab subcutaneously every week (QW) and could transition to Q2W dosing schedule when a ≥ CR was maintained for at least 6 months. Participants could change to Q4W dosing if they achieved CR or better at Cycle 12 Day 1 or later and had been on Q2W dosing for a minimum of 6 months. A comprehensive description of the study design and methods has been previously presented [18, 19].

The study was performed in accordance with the principles of the Declaration of Helsinki and the International Council for Harmonization Good Clinical Practice guidelines. The study protocol and all subsequent amendments were approved by the independent ethics committee at each participating center. Written informed consent was obtained from all participants prior to study entry.

### 2.2 Statistics

The clinical cutoff (CCO) date was September 27, 2024, with a median follow‐up of 27.2 months. A total of 26 participants in the China cohort received at least 1 dose of teclistamab at the RP2D on or before the CCO and were included in the all‐treated analysis set. The responses were assessed per International Myeloma Working Group (IMWG) 2016 criteria [20]. MRD negativity at 10^−5^ testing threshold was assessed using next‐generation flow cytometry. Assessment of PROs included European Organization for Research and Treatment of Cancer Quality of Life Questionnaire Core‐30 Item (EORTC‐QLQ‐C30) [21, 22]. Event rates for binary outcomes were reported together with two‐sided exact 95% confidence intervals (CIs). Time‐to‐event outcomes were analyzed using the Kaplan–Meier approach. Continuous safety data were summarized descriptively using the number of observations, mean, standard deviation, median, and range (minimum and maximum). Categorical data were summarized as counts and corresponding percentages.

## 3 Results

### 3.1 Participant and Treatment

**FIGURE 1:**
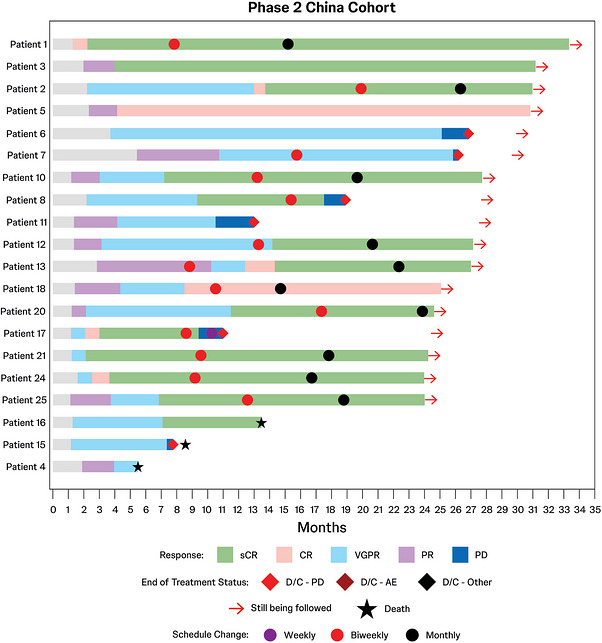
Response and follow‐up based on investigator assessment in all‐treated analysis set. AE, adverse event; CR, complete response; D/C, discontinued; PD, progressive disease; PR, partial response; sCR, stringent complete response; VGPR, very good partial response.

A total of 26 participants were enrolled in the China cohort, and all of them received teclistamab at the RP2D (all‐treated analysis set). The median (range) age was 66 (42–84) years, with 7.7% (*n* = 2) aged ≥ 75 years. Here, 72.2% (*n* = 18) participants had an ECOG‐PS ≥ 1. High‐risk cytogenetics (del[17p], t[4:14], t[14;16]) were present in 57.7% (*n* = 15) participants, and 34.6% (*n* = 9) had ≥ 1 extramedullary disease (EMD) involving soft‐tissue plasmacytoma not associated with bone. Around 23.1% (*n* = 6) of participants had a Revised International Staging System (R‐ISS) Stage III. The median (range) number of prior LOT were 5 (3–11), 61.5% (*n* = 16) were triple‐class refractory. The median (range) follow‐up was 27.2 months (0.8–33.4), and the duration of treatment was 10.1 months (0.7–21.3). Overall, 50% (*n* = 13) participants switched to 1.5 mg/kg Q2W dosing. The median (range) time to switch to Q2W dosing was 12.6 (7.9–20.0) months. In addition, 10/13 (83.3%) participants further switched from Q2W to Q4W dosing and remained in response; the median time to switch from Q2W to Q4W was 6.9 months; participant‐level switch timing is provided in Figure 1.

### 3.2 Efficacy

The ORR rate (95% CI) was 76.9% (56.4–91.0); among responders, 100% achieved ≥ VGPR and 75.0% achieved ≥ CR (Figure 1). Through the CCO of September 27, 2024, the MRD negativity rate at 10^−5^ threshold was 18/26 (69.2%) in all‐treated participants. Among evaluable participants, MRD negativity at 10^−5^ was achieved in 18/20 (90%). Sustained MRD negativity was observed in 9 participants for ≥ 6 months and in 6 participants for ≥ 12 months.

**FIGURE 2:**
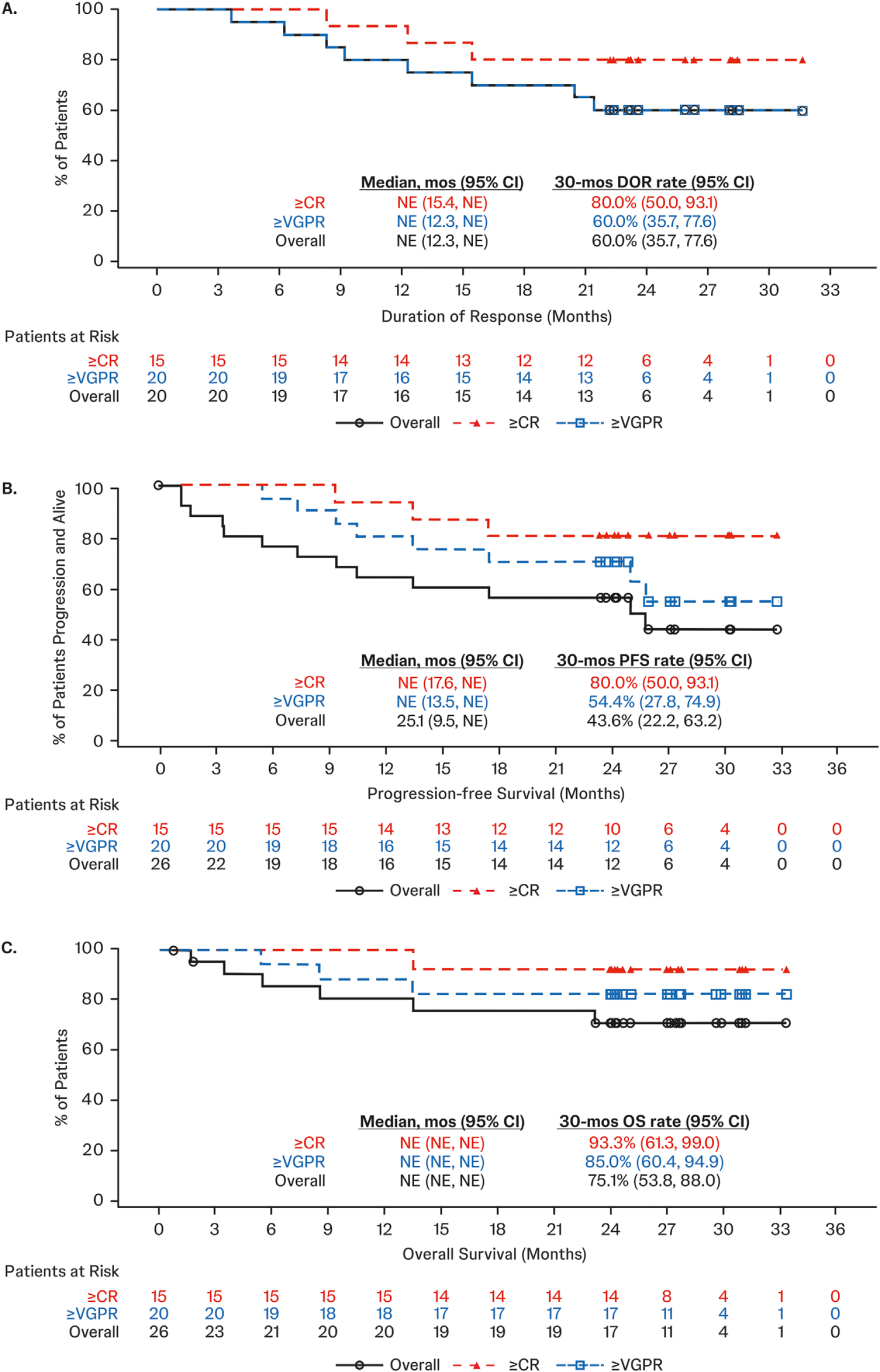
Kaplan–Meier plot for (A) duration of response, (B) progression‐free survival, (C) overall survival in all‐treated analysis set. Response and progression were assessed by investigator, based on IMWG consensus criteria (2016). Responders with schedule change are a subset of the China cohort responders. CI, confidence interval; CR, complete response; DOR, duration of response; IMWG, International Myeloma Working Group; mos, months; NE, not estimable; OS, overall survival; PFS, progression free survival; VGPR, very good partial response.

Across the whole cohort, median (95% CI) DOR was not reached (12.3–NE) (Figure 2a). At 24 months, the estimated DOR rate (95% CI) was 60.0% (35.7–77.6), and this rate remained consistent at 30 months. Median (95% CI) DOR was not reached (12.3–NE) (Figure 2a). Median (95% CI) PFS was 25.1 months (9.5–NE), the estimated PFS rate (95% CI) was 56.0% (34.8–72.7) at 24 months and 43.6% (22.2–63.2) at 30 months (Figure 2b). Median (95% CI) OS was also not reached, and the estimated OS rates (95% CI) were 75.1% (52.8–88.0) at 24 months and 75.1% (53.8–88.0) at 30 months (Figure 2c). Among participants achieving ≥ VGPR, median DOR (NE [12.3, NE]), PFS (NE [13.5, NE]), and OS (NE [NE, NE]) were not reached. The estimated 30‐month DOR, PFS, and OS rates (95% CI) were 60% (35.7, 77.6), 54.4% (27.8, 74.9), and 85% (60.4, 94.9), respectively. Participants achieving ≥ CR experienced longer DOR, PFS, and OS. Median DOR (NE [15.4, NE]), PFS (NE [17.6, NE]), and OS (NE [NE, NE]) were all not reached. At 30 months, the estimated DOR, PFS, and OS rates (95% CI) were 80.0% (50.0–93.1), 80.0% (50.0–93.1), and 93.3% (61.3–99.0).

**FIGURE 3:**
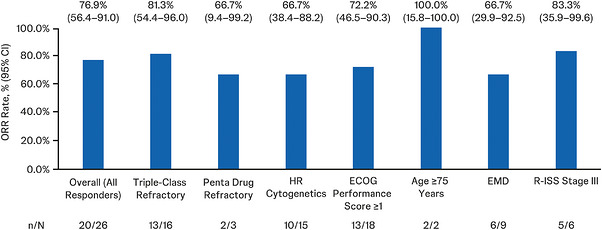
ORR in patients with poor prognostic features. CI, confidence interval; ECOG, Eastern Cooperative Oncology Group; EMD, extramedullary disease; HR, high‐risk; NE, not estimable; ORR, overall response rate; R‐ISS, Revised International Staging System.

Subgroup analyses showed consistent efficacy in subgroups with poor prognostic features. The ORR ranged from 66.7% to 100% in participants with triple‐class or penta‐drug refractory, R‐ISS Stage III disease, high‐risk cytogenetics, aged ≥ 75 years, Eastern Cooperative Oncology Group‐Performance Status (ECOG‐PS) ≥ 1, and EMD involving soft‐tissue plasmacytomas not associated with bone (Figure 3). DOR was also broadly comparable to the overall China cohort across most subgroups, including triple‐ class or penta‐drug refractory, aged ≥ 75 years and ECOG‐PS ≥ 1.

### 3.3 PROs

**FIGURE 4:**
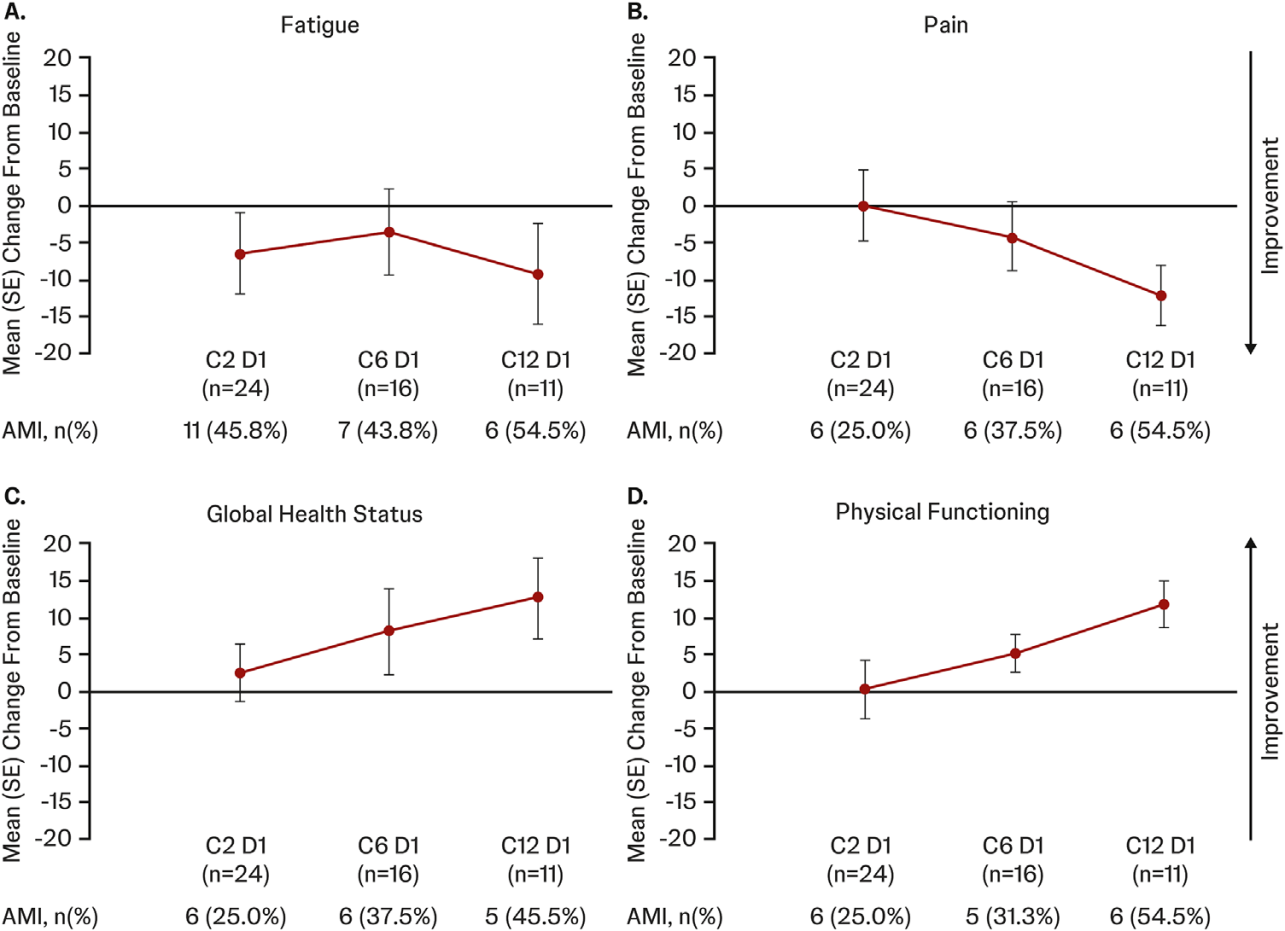
Summary of EORTC‐QLQ‐C30 assessment and change from baseline over time scales in all‐treated analysis set. (A) Fatigue. (B) Pain. (C) Global health status. (D) Physical functioning. All the scores are presented in the range of 0–100 after linear transformation from raw scores (in the range of 1–4). A higher score indicates better health on the global health and functional scales (physical, role, emotional, cognitive, and social) and greater symptom severity on the symptom scales (fatigue, nausea/vomiting, pain, dyspnea, sleep disturbance, appetite loss, constipation, and diarrhea). For literature MCT, 10 points is used while determining AMI. AMI, achieved meaningful improvement; C, cycle; D, day; EORTC‐QLQ‐C30, European Organization for Research and Treatment of Cancer Quality of Life Questionnaire Core‐30 Item; MCT, meaningful change threshold; SE, standard error.

Teclistamab treatment was associated with EORTC‐QLQ‐C30 improvements from Cycle 2 to 12 in global health status scores (mean [standard error; SE]: 2.8 [3.91] to 12.9 [5.55]), physical functioning (0.5 [3.95] to 11.9 [3.18]), fatigue (−6.5 [5.51] to −9.1 [6.84]), and pain (0.0 [23.6] to −12.1 [3.95]) (Figure 4). The median time for improvement ranged from 1.3 to 3.5 months. At Cycle 12, clinically meaningful improvements (≥ 10 points change from baseline [23]) were reported in 45.5% participants for global health status and 54.5% each for physical functioning, fatigue, and pain scores each. Improvements were sustained across subsequent cycles, with the highest rates observed for all scales at Cycle 12 (Figure 4).

### 3.4 Safety

The safety profile of teclistamab was consistent with the known profile of this treatment during the extended follow‐up [16]. With a median follow‐up of 27.2 months, treatment‐emergent adverse events (TEAE) (in ≥ 20% participants) were lymphopenia (100%), neutropenia (100%), cytokine release syndrome (CRS; 96.2%), and leukopenia (96.2%) (Table S1). At least one Grade 3 or 4 TEAE was reported in all 26 participants (100.0%). The pattern and nature of Grade ≥ 3 TEAEs were consistent with those observed in the initial 15‐month analysis [16]. Importantly, no participants discontinued teclistamab or required dose reductions due to TEAEs. Grade 3 or 4 TEAEs occurring in ≥ 20% of participants were predominantly hematologic (lymphopenia [88.5%], neutropenia [76.9%], leukopenia [65.4%], anemia [53.8%], thrombocytopenia [26.9%]), and infections and infestations (COVID‐19 [53.8%], pneumonia [38.5%]), Table 1. Serious TEAEs were reported in 20 participants (76.9%); primarily due to infections and infestations in 18 participants (69.2%), including COVID‐19 (*n* = 15 [57.7%]), pneumonia (*n* = 10 [38.5%]), respiratory failure and bacterial pneumonia (*n* = 3 [11.5%] each), and other events such as upper respiratory tract infection, neutropenia, thrombocytopenia, and diarrhea (*n* = 2 [7.7%] each). Grade 5 TEAEs included multiple organ dysfunction syndrome, cardiac failure, and pneumonia (*n* = 1 [3.8%] each).

**TABLE 1:** Most commonly reported Grade 3 or 4 TEAEs (≥ 5%) in all‐treated participants in MajesTEC‐1 China cohort.

**Grade 3 or 4 TEAEs**	***N* = 26, *n* (%)**
Blood and lymphatic system disorders	26 (100.0%)
Lymphopenia	23 (88.5%)
Neutropenia	20 (76.9%)
Leukopenia	17 (65.4%)
Anemia	14 (53.8%)
Thrombocytopenia	7 (26.9%)
Infections and infestations	20 (76.9%)
COVID‐19	14 (53.8%)
Pneumonia	10 (38.5%)
Pneumonia bacterial	4 (15.4%)
Upper respiratory tract infection	4 (15.4%)
Metabolism and nutritional disorders	12 (46.2%)
Hypokalemia	9 (34.6%)
Hyperglycemia	3 (11.5%)
Hypophosphatemia	3 (11.5%)
Hyponatremia	2 (7.7%)
Gastrointestinal disorders	3 (11.5%)
Diarrhea	3 (11.5%)
Investigations	3 (11.5%)
Blood phosphorus decreased	2 (7.7%)
Respiratory, thoracic, and mediastinal disorders	3 (11.5%)
Respiratory failure	3 (11.5%)
Vascular disorders	3 (11.5%)
Hypertension	2 (7.7%)

*Note*: AEs are coded using MedDRA Version 24.0. The output includes the diagnosis of CRS and ICANS; the symptoms of CRS or ICANS are excluded. AEs are reported until 30 days after the last dose of teclistamab or until the start of subsequent anticancer therapy, if earlier. Participants are counted only once for any given event, regardless of the number of times they actually experienced the event.

Abbreviations: AEs, adverse events; COVID, coronavirus disease; CRS, cytokine release syndrome; ICANS, immune effector cell‐associated neurotoxicity syndrome; MedDRA, medical dictionary for regulatory activities; TEAE, treatment‐emergent adverse event.

With 27.2 months median follow‐up, the rates of CRS remained unchanged [16]. No Grade ≥ 3 neurologic events were observed, and no cases of immune effector cell‐associated neurotoxicity syndrome (ICANS) were observed throughout the study.

### 3.5 Infection and IgG Management

**FIGURE 5:**
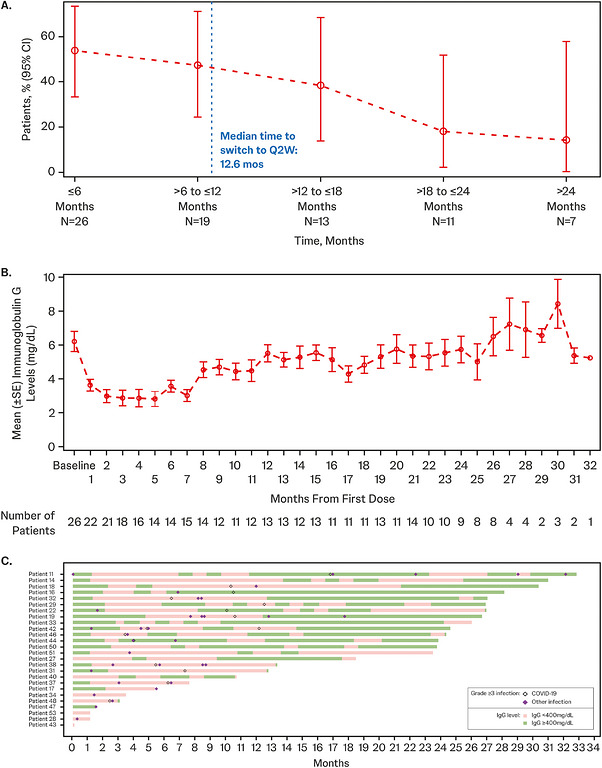
Trends in infection rates and IgG levels over time in the all‐treated analysis set, including (A) new‐onset grade ≥ 3 infections, (B) mean (±SE) IgG levels, and (C) a timeline of grade ≥ 3 infections (COVID‐19 or others) alongside IgG Levels. Includes participants either treated or experienced any TEAE of infections/infestations within the specific window. Percentages are calculated with the number of participants treated within each window as denominator. Here, 95% exact CIs are displayed. AEs are reported until 30 days after the last dose of teclistamab or until the start of subsequent anticancer therapy, if earlier. In cases of IgG myeloma, the M‐spike component was subtracted from the total IgG level to approximate the amount of polyclonal and functional IgG. The highest IgG was presented within each month. AEs, adverse events; CI, confidence interval; COVID‐19, coronavirus disease 2019; IgG, immunoglobulin G; mos, months; Q2W, every 2 weeks; SE, standard error; TEAE, treatment‐emergent adverse events.

A total of 25 participants (96.2%) experienced infections of any‐grade, including Grade 3 or 4 infections in 20 participants (76.9%). Grade 3 or 4 Coronavirus disease 2019 (COVID‐19) occurred in 14 participants (53.8%). A reduction in new‐onset Grade 3 or 4 infections was observed over time, consistent with disease control and temporally coincided with the switch to 1.5 mg/kg Q2W dosing. Grade 3 or 4 infections were reported in 14/26 participants (53.8%) during the first 6 months of treatment, 9/19 participants (47.4%) during > 6–12 months, 5/13 participants (38.5%) during > 12–18 months, 2/11 participants (18.2%) during > 18–24 months, and 1/7 participants (14.3%) beyond 24 months (Figure 5a).

Hypogammaglobulinemia, defined by TEAE reporting and/or ≥ 1 post‐baseline IgG level < 400 mg/dL, was observed in 24/26 participants (92.3%) following teclistamab therapy; median time to onset was 1.4 (Range: 1.1–4.8) months. A total of 22 participants (84.6%) received ≥ 1 dose of intravenous immunoglobulin (IVIg) replacement. Mean IgG level began to rise over the first 6 months of teclistamab therapy and remain consistently > 400 mg/dL after 8 months with ongoing IVIg replacement (Figure 5b). The decline in the incidence of infections over time temporally coincided with the switch to Q2W dosing. Increasing awareness of infection management and use of IVIg to maintain serum IgG levels may also have contributed to this trend. Most Grade 3 or 4 infection events occurred during periods when IgG levels were < 400 mg/dL (Figure 5c).

## 4 Discussion

After more than 2 years of median follow‐up, teclistamab continued to demonstrate clinically meaningful benefit in TCE‐RRMM patients, corroborating the efficacy and safety profiles observed in the earlier 15‐month analysis [16]. At a median follow‐up of 27.2 months, the median DOR had not yet reached, indicating that responses to teclistamab were durable—even among patients with ≥ CR who transitioned to Q2W or Q4W dosing schedules. Teclistamab also demonstrated a median PFS of 25.1 months. At the time of the analysis, the upper bound of the 95% CI for median PFS was not estimable. Notably, patients achieving ≥ CR (representing 75.0% of responders) showed longer DOR, PFS, and OS compared with those who achieved < CR. These findings, together with the results from the MajesTEC‑1 pivotal cohort, reinforce that teclistamab continues to deliver deep and durable responses in heavily pretreated RRMM patients [17].

Efficacy outcomes (e.g., ORR, ≥ CR, mPFS) appeared numerically higher in the MajesTEC‐1 China cohort compared with the MajesTEC‐1 pivotal cohort; however, these findings should be interpreted with caution given the small sample size. Baseline differences may also have contributed, including fewer patients aged ≥ 75 years, fewer penta‐exposed patients, and fewer patients with prior autologous stem cell transplantation in the China cohort. At the same time, the China cohort included a higher proportion of patients with high‐risk cytogenetics and EMD, supporting the robustness of the observed efficacy.

Consistent with the MajesTEC‑1 pivotal cohort, teclistamab demonstrated robust activity across poor prognostic subgroups, including patients with high‑risk cytogenetics, EMD, R‐ISS Stage III disease, those who were triple‐class or penta‐drug refractory, and patients aged ≥ 75 years. In the MajesTEC‑1 China cohort, these subgroups achieved ORRs ranging from 66.7% to 100%, with generally comparable DORs. Nevertheless, patients with high‐risk cytogenetics or EMD exhibited lower 24‐month DOR rates compared with other poor prognostic subgroups. The findings from this study are also supported by emerging real‐world evidence. For example, a multicenter retrospective study of 385 patients (22% aged ≥ 75 years) showed no age‐related differences in rates of ORR and survival outcomes between patients aged ≥ 75 years and those < 75 years [24]. Collectively, these data reinforce the therapeutic potential of teclistamab across diverse patient subgroups, including those with poor prognostic features or limited treatment options.

In our analysis, PROs measured by the EORTC‑QLQ‑C30 demonstrated sustained improvements in global health status, physical functioning, fatigue, and pain among patients treated with teclistamab. These observations align closely with findings from the MajesTEC‑1 pivotal cohort, reinforcing the consistency of health‐related quality‐of‐life (HRQoL) benefits across studies. Notably, post hoc analyses from the MajesTEC‑1 pivotal cohort further indicated that patients achieving ≥ CR experienced more pronounced HRQoL improvements compared with those with less deep responses [25]. These results are particularly relevant in the RRMM setting, where disease‐related symptoms and treatment‐associated toxicities frequently compromise quality‐of‐life (QoL) [26, 27]. By inducing deep and durable responses, teclistamab may mitigate disease burden and its associated symptoms, thereby translating effective biological disease control into meaningful symptomatic and functional improvements.

With longer follow‐up, the safety profile of teclistamab remained largely consistent with that observed in the MajesTEC‑1 pivotal cohort. Although Grade 3/4 infections were more frequent than in the MajesTEC‐1 pivotal cohort, comparisons are limited by the small size of the China cohort (*n* = 26). In addition, Grade 3/4 COVID‐19 (53.8%) accounted for a substantial proportion of Grade 3/4 infections (76.9%) in this cohort, which may have increased the overall incidence of Grade 3/4 infections. We also observed that the reduction in Grade ≥ 3 infections may reflect increased use of IVIg to maintain IgG levels. Infection risk was managed with close clinical/laboratory monitoring and supportive care per protocol and institutional standards, including anti‐infective prophylaxis when appropriate and prompt evaluation and treatment of suspected infections. Guidance for IVIg replacement in patients with hypogammaglobulinemia was in place from the start of the study and was reinforced through subsequent protocol amendments. Accordingly, IVIg replacement was administered to maintain serum IgG ≥ 400 mg/dL; 22/26 (84.6%) patients received IVIg. Mean IgG levels increased over time with IVIg use, began to rise after 6 months of teclistamab therapy, and remained consistently ≥400 mg/dL after 8 months with ongoing IVIg replacement. The incidence of new‐onset Grade ≥ 3 infections decreased later during treatment, temporally coinciding with transition to less‐frequent dosing and improved IgG maintenance. These observations are in line with data showing that IVIg use was associated with a significantly lower risk of serious infections among patients treated with teclistamab [28] and with IMWG guidance endorsing IVIg supplementation [29]. Accordingly, heightened surveillance for infection and prophylaxis IVIg replacement should be considered for patients receiving teclistamab.

Teclistamab was administered at 1.5 mg/kg QW, with transition to Q2W dosing permitted only after a sustained ≥ CR for at least 6 months, as defined by the study protocol. Modeling analyses have suggested that maintaining dose intensity is important to preserve a sufficient effector‐to‐target ratio for continued cell killing until disease burden is reduced [30]. In the MajesTEC‐1 pivotal cohort, 46% of patients achieved ≥ CR, and DOR, PFS, and OS were further improved for patients who achieved ≥ CR than < CR, supporting that deep responses were associated with the most durable clinical benefit [22]. Given the need to balance efficacy with long‐term treatment burden, strategies to reduce dosing frequency while maintaining efficacy are being actively explored. In the ongoing MajesTEC‐9 study, participants receive two step‐up doses followed by 1.5 mg/kg QW for the first two cycles; from Cycles 3–6, dosing transitions to 3 mg/kg Q2W, or 3 mg/kg Q4W in patients achieving ≥ VGPR; from Cycle 7 onward, all participants receive 3 mg/kg Q4W [31]. This strategy aims to improve convenience and flexibility while preserving clinical benefit.

Aside from teclistamab, several studies evaluating BCMA BsAbs in Phase I dose‐escalation and dose‐expansion have been reported. Among BCMA BsAbs currently under investigation in Chinese patients including CM336, GR1803, F182112, and EMB‑06—ORRs were ranged from 39.5% to 85%; however, published data remains limited regarding the depth of response and long‐term clinical outcomes. In terms of adverse events (AEs), GR1803 has shown an incidence of CRS (89.6%) and Grade ≥ 3 CRS (6.3%), and the incidence of Grade ≥ 3 tumor lysis syndrome was 17.4%–41.7%. EMB‑06, a novel Fabs‐in‐tandem immunoglobulin (FIT‐Ig) molecule consisting of 2 + 2 Fab arms designed to modulate binding affinities and potentially reduce CRS, and exhibited a CRS rate of 61.5% within the targeted dose range of 120–300 mg [32]. Although these early data suggest antitumor activity, further follow‐up is needed to better define depth of response, durability, and longer‐term safety across these emerging BCMA BsAbs.

## 5 Conclusions

In the China cohort of the MajesTEC‐1 study, teclistamab continued to demonstrate sustained efficacy and a consistent safety profile in heavily pretreated patients with triple‐class‐exposed RRMM. Over a median follow‐up of 27.2 months, patients achieved deep and durable responses, with high ≥ CR and MRD negativity rates translating into encouraging long‐term outcomes. Early and persistent improvements in PROs further indicated meaningful QoL benefits. Safety findings remained consistent with reported literature, with no treatment discontinuations due to TEAEs. Importantly, the incidence of new‐onset Grade ≥ 3 infections decreased over time, consistent with the time to switch to Q2W dosing and likely, improved awareness and increased adoption of IgG replacement. Overall, these results reinforce teclistamab as an effective and manageable SOC for heavily pretreated RRMM patients in China.

## Author Contributions

**Zhen Cai**: investigation, resources. **Zhongjun Xia**: investigation, resources. **Aili He**: investigation, resources. **Yujun Dong**: investigation, resources. **Yafei Wang**: investigation, resources. **Aijun Liao**: investigation, resources. **Yang Song**: conceptualization, formal analysis, investigation. **Hongye Chen**: conceptualization, formal analysis, investigation. **Athena Zuppa**: conceptualization, formal analysis, investigation. **Katherine Chastain**: conceptualization, formal analysis, investigation. **Latisha Watkins**: conceptualization, formal analysis, investigation. **Xinchao Luo**: conceptualization, formal analysis, investigation. **Lin Huang**: conceptualization, formal analysis, investigation. **Hongmei Xu**: conceptualization, formal analysis, investigation. **Longen Zhou**: conceptualization, formal analysis, investigation**. Wannan Chen**: conceptualization, formal analysis, investigation. **Angeline Zhu**: conceptualization, formal analysis, investigation. **Xiaohong Wang**: conceptualization, formal analysis, investigation. **Weijun Fu**: investigation, resources. **Ting Niu**: investigation, resources. **Juan Du**: investigation, resources.

## Ethics Statement

The study adhered to the principles of the Declaration of Helsinki and the Good Clinical Practice guidelines of the International Council for Harmonization. The study protocol and amendments were approved by the Independent Ethics Committee at every study site.

## Consent

Informed consent was obtained from each participant before enrollment, after being advised of the potential risks and benefits of the study, as well as the investigational nature of the study.

## Conflicts of Interest

Juan Du and Weijun Fu conducted and completed this study at Shanghai Changzheng Hospital. Currently, they are working at Renji Hospital and Shanghai Fourth People's Hospital, respectively. Yang Song, Katherine Chastain, Latisha Watkins, Xinchao Luo, Lin Huang, Hongye Chen, Athena Zuppa, Hongmei Xu, Longen Zhou, Wannan Chen, Angeline Zhu, and Xiaohong Wang are employees of, and may own stock/stock options in Johnson & Johnson. The other authors declare no conflicts of interest.

## Supporting information




**Supporting Information**: jha270340‐sup‐0001‐SuppMat.docx

## Data Availability

The data sharing policy of Johnson & Johnson is available at https://innovativemedicine.jnj.com/our‐innovation/clinical‐trials/transparency.
