# Supplementary material for: Efficacy and Safety of Teclistamab in Relapsed/Refractory Multiple Myeloma: 2‐Year Follow‐Up From MajesTEC‐1 China Cohort
Source: EJHaem. 2026 Jul 30;7(4):e70340. doi: 10.1002/jha2.70340 (PMC13421914; doi:10.1002/jha2.70340)
Supplement: Supplementary file 1 — Supporting Information: jha270340‐sup‐0001‐SuppMat.docx [file JHA2-7-e70340-s001.docx]

[Supplementary Material]

**Supplementary Table S1. Most commonly reported TEAEs (≥20%) in all-treated participants in MajesTEC-1 China cohort.**

| Commonly reported TEAEs (≥20%) | N=26, n (%) |
| --- | --- |
| Blood and lymphatic system disorders | 26 (100.0) |
| Lymphopenia | 26 (100.0) |
| Neutropenia | 26 (100.0) |
| Leukopenia | 25 (96.2) |
| Anemia | 23 (88.5) |
| Thrombocytopenia | 15 (57.7) |
| Hyperglobulinemia | 8 (30.8) |
| Immune system disorders | 25 (96.2) |
| Cytokine release syndrome | 25 (96.2) |
| Hypogammaglobulinemia | 14 (53.8) |
| Infections and infestations | 25 (96.2) |
| COVID-19 | 19 (73.1) |
| Pneumonia | 11 (42.3) |
| Upper respiratory tract infection | 11 (42.3) |
| Metabolism and nutritional disorders | 25 (96.2) |
| Hypoalbuminemia | 20 (76.9) |
| Hypokalemia | 20 (76.9) |
| Hypocalcemia | 15 (57.7) |
| Hypertriglyceridemia | 11 (42.3) |
| Hypomagnesaemia | 10 (38.5) |
| Hypophosphatemia | 8 (30.8) |
| Hyperuricemia | 7 (26.9) |
| Hyponatremia | 7 (26.9) |
| Hyperglycemia | 6 (23.1) |
| Gastrointestinal disorders | 23 (88.5) |
| Diarrhea | 15 (57.7) |
| Constipation | 7 (26.9) |
| Toothache | 6 (23.1) |
| Investigations | 22 (84.6) |
| Blood lactate dehydrogenase increased | 11 (42.3) |
| Aspartate aminotransferase increased | 10 (38.5) |
| Alanine aminotransferase increased | 9 (34.6) |
| Gamma-glutamyl transferase increased | 6 (23.1) |
| General disorders and administration site conditions | 20 (76.9) |
| Injection site erythema | 6 (23.1) |
| Injection site rash | 6 (23.1) |
| Musculoskeletal and connective tissue disorders | 17 (65.4) |
| Respiratory, thoracic, and mediastinal disorders | 14 (53.8) |
| Cough | 10 (38.5) |
| Productive cough | 6 (23.1) |
| Nervous system disorders | 13 (50.0) |
| Headache | 8 (30.8) |
| Vascular disorders | 8 (30.8) |
| Psychiatric disorders | 6 (23.1) |
| Insomnia | 6 (23.1) |
| Skin and subcutaneous tissue disorders | 6 (23.1) |

AEs are coded using MedDRA Version 24.0. AEs are reported until 30 days after the last dose of teclistamab or until the start of subsequent anticancer therapy, if earlier. Participants are counted only once for any given event, regardless of the number of times they actually experienced the event. Percentages calculated with the number of participants in all-treated analysis set as denominator.

AEs, adverse events; COVID, coronavirus disease; MedDRA, medical dictionary for regulatory activities; TEAE, treatment-emergent adverse event.
